# Pullulan Production by *Aureobasidium pullulans* ATCC 201253 Cells Adsorbed onto Cellulose Anion and Cation Exchangers

**DOI:** 10.5402/2012/140951

**Published:** 2012-09-23

**Authors:** Thomas P. West

**Affiliations:** Department of Biology and Microbiology, South Dakota State University, Brookings, SD 57007, USA

## Abstract

The anion exchanger phosphocellulose and the cation exchanger triethylaminoethyl cellulose were used to immobilize cells of the fungus *Aureobasidium pullulans* ATCC 201253 and the adsorbed cells were subsequently investigated for their ability to produce the polysaccharide pullulan using batch fermentation. The cells adsorbed on the triethylaminoethyl cellulose at pH 7.5 produced higher pullulan levels than those cells immobilized on phosphocellulose at pH 4.0 for 2 cycles of 168 h at 30 °C. Relative to the initial cycle of 168 h, pullulan production by the cells immobilized on the triethylaminoethyl cellulose decreased slightly after 168 h of the second production cycle while pullulan production by the phosphocellulose-immobilized cells remained about the same after 168 h of the second production cycle.

## 1. Introduction

Pullulan is an extracellular polysaccharide synthesized by the fungus *Aureobasidium pullulans* utilizing an excess of carbon with limiting nitrogen present [1–4]. The polysaccharide consists of primarily cross-linked maltotriose although a small proportion of maltotetraose residues has been detected in its structure [5–9]. Due to its high water solubility and low viscosity, pullulan has numerous commercial applications including its use as a food additive, a flocculant, a blood plasma substitute, an adhesive, and a film [8, 10, 11].

 Immobilization of *A*.* pullulans* cells by adsorption onto solid supports has been examined in previous studies. The advantage of using immobilized cells is that they can be used for more than a single cycle of polysaccharide production. When *A. pullulans* ATCC 42023 cells were immobilized by adsorption onto the solid support diatomaceous earth, the immobilized cells produced pullulan and were capable of being reutilized for a second cycle of pullulan production [12, 13]. Similarly, *A*. *pullulans* ATCC 42023 cells adsorbed onto sponge cubes were capable of several cycles of polysaccharide production [14]. Entrapped cells of *A*. *pullulans* can also be utilized to produce pullulan for at least two production cycles [15, 16]. A biofilm reactor using a plastic composite support found that pullulan production by immobilized *A*. *pullulans* ATCC 201253 cells was maximal at pH 5.0 after 168 h [17]. Ion exchange resins, such as DEAE (diethylaminoethyl) cellulose and ECTEOLA (epichlorohydrin triethanolamine) cellulose, have been utilized to adsorb corn syrup-grown cells of *A*. *pullulans* ATCC 42023, and the adsorbed cells produced pullulan for two production cycles [13, 18, 19]. The cells of the reduced pigmentation mutant strain *A*. *pullulans* ATCC 201253 have been adsorbed onto ion exchange resins and investigated relative to pullulan production [18, 20]. It was found that ATCC 201253 cells could be adsorbed at pH 2.0 onto ion exchangers to produce pullulan from corn syrup [18].

 In this study, pullulan production by corn syrup-grown cells of *A*. *pullulans* ATCC 201253 immobilized by adsorption onto the anion exchange resin phosphocellulose at pH 4.0 or the cation exchange resin TEAE (triethylaminoethyl) cellulose at pH 7.5 using batch fermentation was compared. The effectiveness of utilizing the fungal cells adsorbed onto the ion exchangers for more than one cycle of batch pullulan production was also compared. 

## 2. Materials and Methods

### 2.1. Strain and Media

*Aureobasidium pullulans* ATCC 201253 was the strain used in this work [20]. The composition of the culture medium was previously described [21]. In the phosphate-buffered medium, corn syrup served as the carbon source at a final concentration of 2.5% (w/v) while ammonium sulfate served as the nitrogen source at a final concentration of 0.06% (w/v). After inoculating batch cultures (50 mL) with overnight cultures (0.5 mL) grown in the same culture medium, each batch culture was shaken at 200 rpm for 48 h at 30°C.

### 2.2. Cell Immobilization on Ion Exchangers

Phosphocellulose or TEAE cellulose (1 g) was pretreated by sequentially washing with 200 mL of 0.25 N HCl and 0.25 N NaOH in a sterile 250 mL Erlenmeyer flask. The resins were then resuspended in 0.5 N HCl (200 mL) overnight to sterilize them [22, 23]. Following washing the resins at least three times with sterile water (200 mL), the water was removed from the resins and the resins could be utilized for cell adsorption. Phosphocellulose was suspended in 50 mL of culture medium (pH 4.0) containing 2.5% (w/v) corn syrup while the TEAE cellulose was suspended in 50 mL of culture medium (pH 7.5). After each suspension was inoculated with medium containing about 10^5^ fungal cells/mL (from 48 h batch cultures), each flask was shaken (100 rpm) for 48 h at 30°C. Each ion exchange resin with the adsorbed fungal cells was collected by low-speed centrifugation and washed twice with 0.85% NaCl. After each resin was suspended in 50 mL of culture medium (pH 6.0) containing 2.5% (w/v) corn syrup, the immobilized cells in the flasks were shaken (125 rpm) for 168 h at 30°C. Pullulan production by fungal cells immobilized at pH 4.0 on phosphocellulose or at pH 7.5 on TEAE cellulose was monitored at 24 h intervals. Following the first cycle of 168 h, each cellulosic ion exchanger was collected by low-speed centrifugation, washed, and again suspended in culture medium (pH 6.0) containing 2.5% (w/v) corn syrup. The cultures were shaken (125 rpm) for a second cycle of 168 h at 30°C during which the pullulan levels were measured at an interval of 24 h.

### 2.3. Assays and Statistical Analysis

To monitor polysaccharide production, culture medium (2 mL) was removed and the sample was centrifuged (14,600 g, 30 min, 4°C) with the supernatant being used for the pullulan determinations. To one volume of the pullulan-containing supernatant, two volumes of 95% ethanol were added to precipitate the polysaccharide. The precipitated pullulan was collected on 0.45 *μ*m HVLP filters (25 mm diameter). All filters were dried to constant weight at 105°C and reweighed to determine pullulan concentrations [21]. The viable fungal cell concentration of the adsorbed cells on each ion exchange resin was determined as stated previously [18]. Pullulan concentrations were expressed as g/l and represent the mean of three independent determinations. The Student's *t*-test was used during statistical analysis.

## 3. Results and Discussion

Culture medium pH has been shown to affect fungal cell adsorption onto the ion exchange resins DEAE cellulose, phosphocellulose, TEAE cellulose, and ECTEOLA cellulose [13, 18, 19]. The culture medium pH used to adsorb the *A*. *pullulans* ATCC 201253 cells onto the resins was 2.0 which was based upon observed pullulan production by the immobilized cells incubated in medium (pH 6.0) containing 3% corn syrup [13, 19]. In this study, it was determined that a higher concentration of cells could be adsorbed when the culture medium pH was elevated. The concentration of ATCC 201253 cells adsorbed at pH 4.0 onto the phosphocellulose was determined to be 1.4 × 10^5^ ± 0.9 × 10^5^ (mean ± standard deviation) colony-forming units/mg dry weight of resin which is higher than the concentration of 3.2 × 10^4^ ± 0.1 × 10^4^ (mean ± standard deviation) colony-forming units/mg dry weight of resin noted previously for adsorption onto phosphocellulose at pH 2.0 [18]. Similarly, the concentration of ATCC 201253 cells adsorbed at pH 7.5 onto the TEAE cellulose was found to be 5.4 × 10^6^ ± 6.5 × 10^6^ (mean ± standard deviation) colony-forming units/mg dry weight of resin which is higher than the concentration of 1.2 × 10^5^ ± 0.1 × 10^5^ (mean ± standard deviation) colony-forming units/mg dry weight of resin observed previously for adsorption onto TEAE cellulose at pH 2.0 [18]. With the concentration of adsorbed cells being increased by elevating the culture medium pH used for cell adsorption, it was of interest to compare pullulan production by ATCC 201253 cells adsorbed onto phosphocellulose at pH 4.0 and onto TEAE cellulose at pH 7.5.

**Figure 1:**
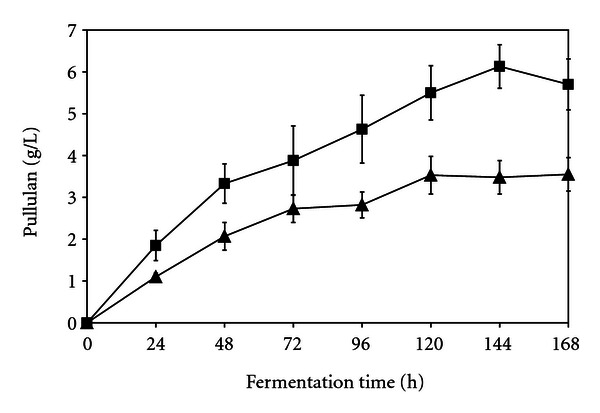
Pullulan levels (g/l) produced over a period of 168 h during cycle 1 by *Aureobasidium pullulans* ATCC 201253 cells adsorbed on triethylaminoethyl cellulose (■) or phosphocellulose (▲). Error bars indicate the standard deviations of mean data values.

**Figure 2:**
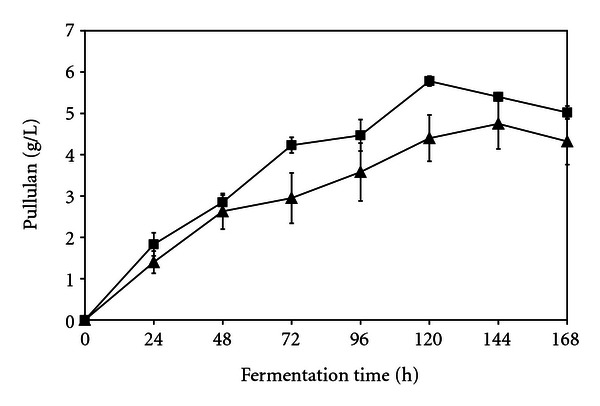
Pullulan levels (g/l) produced over a period of 168 h during cycle 2 by *Aureobasidium pullulans* ATCC 201253 cells adsorbed on triethylaminoethyl cellulose (■) or phosphocellulose (▲). Error bars indicate the standard deviations of mean data values.

 With respect to both ion exchange resins, the ability of the immobilized cells to produce pullulan when incubated in medium (pH 6.0) containing 3% corn syrup during the initial cycle of polysaccharide production for 168 h using batch fermentation was investigated. As can be seen in Figure 1, the cells adsorbed on phosphocellulose produced pullulan after 24 h and continued to produce it for 120 h during the initial cycle of production. Pullulan production by the cells adsorbed on TEAE cellulose was noted after 24 h and continued to be produced for 144 h during the initial cycle of production (Figure 1). Pullulan production by the immobilized cells appeared to increase little after 144 h for cells adsorbed on either ion exchanger (Figure 1). The pullulan levels produced during the initial cycle of 168 h by the cells adsorbed onto TEAE cellulose were higher than the polysaccharide levels produced by the cells adsorbed onto phosphocellulose (Figure 1). The difference in pullulan production after 96 h, 120 h, 144 h, and 168 h between the cells adsorbed onto phosphocellulose at pH 4.0 and the cells adsorbed onto TEAE cellulose at pH 7.5 was statistically significant (*P* < 0.01). As can be seen in Figure 2, pullulan levels produced during the second cycle of 168 h by the cells adsorbed onto TEAE cellulose were higher than the polysaccharide levels produced by the cells adsorbed onto phosphocellulose which was also noted during the initial cycle of production. The difference in pullulan production after 120 h between the cells adsorbed onto phosphocellulose at pH 4.0 and the cells adsorbed onto TEAE cellulose at pH 7.5 was statistically significant (*P* < 0.01). For the cells adsorbed on the phosphocellulose or TEAE cellulose, there was no statistical difference in pullulan production after 168 h between the first and second production cycle. It appeared that pullulan production by the immobilized cells on either ion exchanger was equally effective for two cycles of production for 168 h. The pH 4.0-adsorbed ATCC 201253 cells onto phosphocellulose used in this study produced less polysaccharide than the pH 2.0-adsorbed cells on phosphocellulose during the initial production cycle of 168 h [18]. In contrast, the pH 4.0-adsorbed ATCC 201253 cells used in this work produced higher polysaccharide levels on phosphocellulose than the pH 2.0-adsorbed cells during the second production cycle of 168 h [18]. The pH 7.5-adsorbed ATCC 201253 cells on TEAE cellulose used in this study produced higher polysaccharide levels than the pH 2.0-adsorbed cells on phosphocellulose during both production cycles of 168 h [18].

 Prior studies have investigated the immobilization of *A*. *pullulans* ATCC 42023 cells using the ion exchange resins DEAE cellulose or ECTEOLA cellulose and the ability of the immobilized cells to produce pullulan using batch fermentation [13, 19]. ATCC 42023 cells were adsorbed onto DEAE cellulose or ECTEOLA cellulose at pH 2.0 [13, 19]. Pullulan production by the ATCC 201253 cells adsorbed on TEAE cellulose used in this study was more similar to production by the DEAE cellulose-adsorbed ATCC 42023 cells than the ECTEOLA cellulose-adsorbed ATCC 42023 cells since the second cycle of production was lower than the initial production cycle [13, 19]. Although the findings from this study showed that the phosphocellulose-adsorbed cells produced less polysaccharide than ECTEOLA cellulose-adsorbed cells, pullulan production by the ECTEOLA cellulose-adsorbed cells was similar to pullulan production by the phosphocellulose adsorbed cells because pullulan levels decreased relatively little during the second production cycle compared to the initial production cycle [19].

## 4. Conclusions 

The cation exchanger TEAE cellulose appeared to be a more effective adsorption support to immobilize *A*. *pullulans* ATCC 201253 cells when the cells were adsorbed at pH 7.5 instead of pH 2.0. The anion exchanger phosphocellulose was less effective than TEAE cellulose for pullulan production by ATCC 201253 cells independent of whether adsorption occurred at pH 2.0 or 4.0. The ATCC 201253 cells adsorbed at pH 4.0 onto phosphocellulose were able to produce pullulan at comparable levels during both production cycles unlike the pH 2.0-adsorbed ATCC 201253 cells where pullulan production diminished during the second production cycle. Further study of *A*. *pullulans* cell immobilization using anion and cation exchangers for pullulan production will be necessary to identify the most effective ion exchanger that adsorbs high fungal cell concentrations and produces high pullulan levels for several cycles.
